# Evolution of reproductive life‐history and dispersal traits during the range expansion of a biological control agent

**DOI:** 10.1111/eva.13502

**Published:** 2022-11-01

**Authors:** Eliza I. Clark, Ellyn V. Bitume, Dan W. Bean, Amanda R. Stahlke, Paul A. Hohenlohe, Ruth A. Hufbauer

**Affiliations:** ^1^ Graduate Degree Program in Ecology, Department of Agricultural Biology Colorado State University Fort Collins Colorado USA; ^2^ Pacific Southwest Research Station Institute of Pacific Islands Forestry, USDA Forest Service Hilo Hawaii USA; ^3^ Colorado Department of Agriculture Palisade Insectary Palisade Colorado USA; ^4^ Initiative for Bioinformatics and Evolutionary Studies, Department of Biological Sciences University of Idaho Moscow Idaho USA; ^5^ Bee Research Laboratory USDA, Agricultural Research Service, Beltsville Agricultural Research Center Beltsville Maryland USA

**Keywords:** condition‐dependent dispersal, *Diorhabda carinulata*, expansion load, spatial sorting, tethered flight mill

## Abstract

Evolutionary theory predicts that the process of range expansion will lead to differences in life‐history and dispersal traits between the core and edge of a population. At the edge, selection and genetic drift can have opposing effects on reproductive ability, while spatial sorting by dispersal ability can increase dispersal. However, the context that individuals experience, including population density and mating status, also impacts dispersal behavior. We seek to understand the shifts in traits of populations expanding across natural, heterogenous environments, and the evolutionary and behavioral factors that may drive those shifts. We evaluated theoretical predictions for evolution of reproductive life‐history and dispersal traits using the range expansion of a biological control agent, *Diorhabda carinulata*, or northern tamarisk beetle. We find that individuals from the edge had increased fecundity and female body mass, and reduced age at first reproduction, indicating that genetic load is low and suggesting that selection has acted at the edge. We also find that density of conspecifics during rearing and mating status influence dispersal of males and that dispersal increases at the edge of the range under certain conditions, particularly when males were unmated and reared at low density. The restricted conditions in which dispersal has increased suggest that spatial sorting has exerted weak effects relative to other potential processes. Our results support most theoretical predictions about evolution during range expansion, even across a heterogeneous environment, especially when the ecological context is considered.

## 1 INTRODUCTION

**FIGURE 1:**
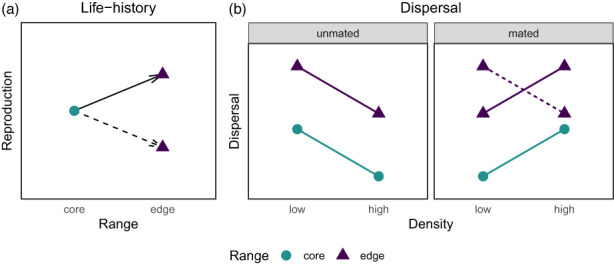
Reproductive life‐history traits in edge populations may increase due to selection at low densities (a, solid arrow) or decrease due to expansion load (a, dashed arrow) compared to core. Dispersal is predicted to be informed by density and mating status and evolves upwards at the range edge (b). Density dependence may also evolve at the edge (b, dashed line).

Ecological and evolutionary processes acting during range expansion (Bowler & Benton, 2005; Kokko & López‐Sepulcre, 2006; Kubisch et al., 2014) are key to understanding the spread of invasive species (Hastings et al., 2005), potential success of biological control agents (Szűcs et al., 2019), and the ability of threatened species to track recent climate change (Mustin et al., 2009). The landscapes encountered by range‐expanding populations represent novel selective environments (Brown et al., 2013; Van Petegem et al., 2016), and simultaneously, the expansion itself can be a catalyst for evolution through spatial sorting and founder effects (Phillips et al., 2010b; Shine et al., 2011). Thus, range expansion can result in evolved differences in reproductive life‐history and dispersal traits between individuals at the core of the range and the edge of the expansion front (Peischl et al., 2013; Phillips, 2015; Simmons & Thomas, 2004). We seek to evaluate such shifts occurring in a range expansion across a natural and heterogenous environment. Through documenting those patterns, and how they align with different theoretical predictions (Figure 1), we then can explore the possible evolutionary processes that may be acting on life‐history and dispersal traits.

Theory predicts that reproductive life‐history traits, such as fecundity and age at first reproduction, can evolve in contrasting ways at the expanding edge relative to the core, depending upon whether selection or nonadaptive evolutionary processes are dominant at the expansion front (Phillips et al., 2010b). When selection is dominant, the edge of an expanding population is predicted to exhibit higher fecundity and earlier age at first reproduction than the core (Burton et al., 2010; Fronhofer & Altermatt, 2015). The stable, high‐density core of a population generally exhibits density‐dependent growth, where selection favors the ability to compete at high densities of conspecifics through having fewer, larger offspring (Phillips et al., 2010b). At the expansion front, population densities are low and competition is relaxed, so the edge generally exhibits density‐independent growth (Altwegg et al., 2013; Burton et al., 2010) and selection favors high fecundity and early reproduction (Figure 1a, solid arrow) (Brommer et al., 2002; Phillips et al., 2010b). Alternatively, when nonadaptive processes are dominant, the edge may experience reduced fitness, or expansion load, relative to the core (Figure 1a, dashed arrow) (Peischl et al., 2013; Peischl & Excoffier, 2015; Travis et al., 2007). This occurs when there are so few individuals at the edge that genetic drift can overwhelm selection, and deleterious alleles may “surf” the wave of expansion during repeated founder events (Klopfstein et al., 2006). Additionally, fecundity at the edge may be reduced relative to the core due to trade‐offs between dispersal, reproduction, and competitive ability (Burton et al., 2010; Fronhofer & Altermatt, 2015; Phillips et al., 2010b). There is model and experimental evidence for both the evolution of increased fecundity at the edge (Phillips, 2009; Siemann & Rogers, 2001) and the evolution of decreased fecundity at the edge (González‐Martínez et al., 2017; Peischl et al., 2013). Insights from range expansions in natural rather than laboratory conditions will help elucidate which processes dominate in nature.

Theory predicts that dispersal will evolve at the expanding edge to increase relative to the core through the process of spatial sorting (Phillips et al., 2008; Shine et al., 2011; Travis & Dytham, 2002). Spatial sorting occurs when individuals with greater dispersal ability arrive at the range edge together and mate with each other. Since dispersal ability is heritable in many species (Saastamoinen et al., 2018), this spatially assortative mating among individuals at the edge produces offspring with even higher dispersal ability. Despite strong evidence for spatial sorting (e.g. Berthouly‐Salazar et al., 2012; Hill et al., 2011; Lombaert et al., 2014; Merwin, 2019; Monty & Mahy, 2010; Phillips et al., 2006, 2010a), some factors may inhibit or weaken evolutionary shifts in dispersal between core and edge. For example, expansion over heterogenous landscapes (like latitudinal changes in climate or host plant genotype) and adaptation to novel environments could slow expansion speed and reduce spatial selection on dispersal (Andrade‐Restrepo et al., 2019; Hillaert et al., 2015). Additionally, species that are unlikely to disperse from low‐density patches may be less likely to evolve increased dispersal ability at the edge of the range expansion (Fronhofer, Gut, & Altermatt, 2017; Travis & Dytham, 2002).

Dispersal is a multi‐faceted behavior that involves individual choices about whether and how far to move. Dispersal may be informed by intraspecific interactions such as the presence of relatives and population density (Bitume et al., 2013; Endriss et al., 2019), and factors internal to the organism such as body condition, sex, or mating status (Figure 1b) (Clobert et al., 2009; Schumacher et al., 1997). For many species, high population density can signal strong intraspecific competition, which may increase emigration (positive density dependence) (Altwegg et al., 2013). Alternatively, species for which the benefit of living near conspecifics (e.g. mate availability, predator avoidance, reduced Allee effects) outweigh the cost of competition may decrease dispersal at high population densities (negative density dependence) (Bowler & Benton, 2005). During range expansion, spatial selection increases dispersal even when population density is low, so density‐dependent dispersal that is less strongly positive, or even negative, may evolve at the range edge (Figure 1b, dashed line) (De Bona et al., 2019; Fronhofer, Nitsche, & Altermatt, 2017; Travis et al., 2009).

Mating status may also influence dispersal decisions for sexually reproducing species that can disperse before and after mating (Clobert et al., 2009; Li & Kokko, 2019; Schumacher et al., 1997). Mated individuals may show positive density‐dependent dispersal to reduce competition and reproduce in a low‐density environment where offspring might have a better chance of survival (Figure 1b, right), while unmated individuals may show negative density‐dependent dispersal and disperse more from low density to increase the chances of finding a mate (Figure 1b, left) (Clobert et al., 2009).

We can infer the relative dominance of evolutionary processes during a natural range expansion by evaluating the patterns of key reproductive life‐history and dispersal traits across the range. We use the range expansion of *Diorhabda carinulata,* an introduced biological control agent (hereafter, biocontrol agent), and examine patterns in life‐history and dispersal traits to evaluate drivers of evolutionary change in range expansions in natural populations. Success of the biocontrol program has provided impetus for research, including this study, focused on the evolutionary and ecological processes enabling beetles to suppress a major invasive plant across western North America (Bean et al., 2012). This study contributes to a growing literature testing range expansion theory on natural populations (Phillips et al., 2006, 2010a; Wolz et al., 2020) and is the first test we know of in a modern biocontrol agent (Szűcs et al., 2019). Understanding the evolutionary dynamics of biocontrol agents is of particular interest for predicting future spread and improving efficacy and safety across the range of a target pest species (Stahlke et al., 2022; Szűcs et al., 2019; Van Klinken & Edwards, 2002; Wright & Bennett, 2018).

**FIGURE 2:**
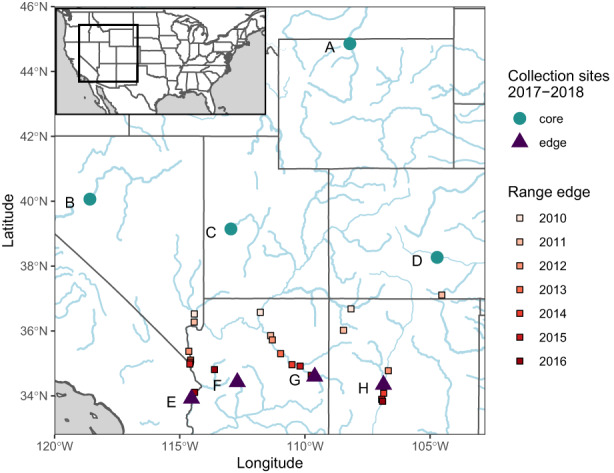
*Diorhabda carinulata* range expansion along river corridors and collection sites at the core and edge (circles and triangles). Squares show edge locations each year, 2010 to 2016, and were jittered to show overlapping points. Letters correspond to Table S1. Range edge data are from the RiversEdge West Tamarisk Beetle Distribution Map, available at: https://arcg.is/1izOPW0 (RiversEdge West, 2021).

*Diorhabda carinulata*, the northern tamarisk beetle, was released in 2001 into the western United States for the biological control of invasive riparian shrubs in the genus *Tamarix*, or saltcedar or tamarisk (DeLoach et al., 2003). Beetles have dispersed southward from a few initial release sites (Figure 2) (Bean et al., 2012) following remote riparian corridors, which likely represent independent dispersal pathways. The *D. carinulata* range expansion provides an excellent study system for testing the predictions of range expansion theory because original release sites are precisely known, the range expansion has been monitored, and expansion along river corridors provides natural spatial replicates of expanding edge collection sites (Bean & Dudley, 2018). We use a common garden to evaluate the patterns of evolution of early fecundity, age at first reproduction, body mass, and dispersal of eight collections of *D. carinulata*, collected from the core and edge of its range in the western US.

Figure 1 summarizes how we can infer the evolutionary processes occurring during range expansion based on the pattern of trait values we find at the core and edge. Specifically, an increase in reproductive output of individuals from the edge relative to the core supports selection at low densities as the dominant process, while decreased reproductive output of edge individuals supports expansion load or trade‐offs as the dominant process (Figure 1a). Increased dispersal among individuals from the edge indicates that spatial sorting is acting during this range expansion (Figure 1b), while if dispersal does not change between core and edge, other factors, such as adaptation to the new environment, may be limiting the opportunity for spatial sorting. We also expect that individuals' dispersal choices will be influenced by the contexts they experience, specifically density and mating status (Figure 1b). If density dependence has evolved during the range expansion, we predict that dispersal will be less positively or negatively density‐dependent for mated individuals from the range edge (Figure 1b, dashed line).

## 2 MATERIALS AND METHODS

*Diorhabda carinulata* are highly specialized leaf‐eating beetles that feed on plants in the genus *Tamarix*. The primary host plants (hybrids between *Tamarix ramosissima* and *Tamarix chinensis*) occupy riparian corridors throughout the area of the *D. carinulata* range expansion, providing abundant habitat for the beetle from range core to edge and beyond. During the summer, male *D. carinulata* emit a pheromone that attracts other beetles and likely assists in mate‐finding (Bean, Wang, et al., 2007; Cossé et al., 2005). It is also common to observe a single beetle on a tree, especially when there are few beetles in an area. In the late summer, adults are cued by shortening daylengths to enter reproductive diapause for the winter (Bean, Wang, et al., 2007). The beetle has adapted to variation in daylength along latitude during this range expansion by shortening the daylength cue so that the timing of diapause remains synchronized with the season at southern latitudes (Bean, Dudley, et al., 2007).

Eight collections of *Diorhabda carinulata* were made from across the introduced range of the species. At least 200 adult beetles were collected by hand at each site, with no more than five beetles being collected from a single tree where possible. Four collections were from well‐established original introduction sites in the north of the range and represent the range core. Another four collections were from the newly established southern edge of the range (Figure 2, Table S1; RiversEdge West, 2021). One edge site (H in Figure 2 and Table S1) was collected slightly behind the edge in 2017 in order to avoid other *Diorhabda* species that were moving northward and overlapping the *D. carinulata* range (Ozsoy et al., 2019). Two sites (one core and one edge) were collected Fall 2017, and the first lab generations were put into reproductive diapause to reduce the number of lab generations before the start of the experiment. All other sites were collected in Summer 2018 and cultured in the lab for one generation to standardize maternal environment effects prior to starting experiments. All insects were reared in growth chambers with a 16 h/8 h light/dark cycle and 25°C/20°C day/night temperatures (reproductive conditions (Bean, Wang, et al., 2007)), and were fed fresh tamarisk as needed.

### 2.1 Life‐history

Newly emerged adult females of the second lab generation were weighed before feeding and reared individually thereafter in 0.24 L plastic containers with mesh lids. Three days after eclosion, each female was paired with a male from the same collection site of about the same age and allowed to mate for 24 h in the same containers as above before the male was removed. Presence of eggs in each container was assessed daily. All eggs that were laid on the first day of oviposition were counted to provide a measure of early fecundity and the age at first reproduction in days since adult emergence was recorded. Individuals that had not oviposited within 7 days of emergence were recorded as nonlayers (Lewis et al., 2003). We collected body mass at eclosion and egg count data from 130 core and 140 edge female *D. carinulata* and age at first reproduction from 104 core and 118 edge females.

### 2.2 Dispersal

Dispersal ability was measured for only male *D. carinulata* since, in the field, males have been observed dispersing first and using pheromones to attract mixed‐sex aggregations of reproductive adults (Cossé et al., 2005). After emergence as adults, males were randomly assigned to mating treatments and density. As described above, both mating status and density experienced can influence dispersal decisions as, for example, unmated beetles might fly in search of mates, or beetles that find themselves in higher densities of conspecifics might fly in search of low‐competition sites. Males assigned to the mated treatment were paired with a female from the same collection site for 24 h. The males were thereafter reared in 0.24 L plastic containers with mesh lids in groups of five (high density) or alone (low density). All males in each high‐density container were of the same mating treatment. All containers received the same surplus amount of fresh tamarisk, regardless of how many beetles were in the container. Density conditions were designed to allow us to estimate how local social interactions will impact decisions to disperse and not intended to simulate density conditions in the field. Male beetles were between 6 and 23 days after eclosion during the dispersal trial and were weighed on the day of the dispersal trial.

We assessed dispersal of male beetles using tethered flight mills (reviewed in Minter et al., 2018), similar in design to Maes et al. (2014) (Appendix S1). Each beetle was given one hour to take any number of flights on a flight mill. Data from each trial were converted into four dispersal elements: occurrence of at least one flight, number of flights, total flight distance, and average flight speed (Appendix S2). Each dispersal element has different biological relevance (Stevens et al., 2013; Tung et al., 2017). Occurrence of flight and number of flights measure the probability and frequency of movement from the local patch. Total distance and average speed measure how far individuals disperse, after initiation. Spatial sorting may act on any one or combination of these elements. We collected dispersal data from 279 core males and 311 edge males, with 65 to 81 males in each density‐mating treatment combination and at least 15 from each population. Average speed was calculated for 231 core and 266 edge males that took at least one flight, with 56 to 71 males in each density‐mating treatment combination.

### 2.3 Statistical analyses

The three life‐history traits of female body mass, fecundity over 24 h, and age at first reproduction were analyzed both individually and with a multivariate analysis of variance (MANOVA). In the MANOVA, only individuals that produced eggs during the experiment were included, since they were the only complete observations. The three life‐history traits were the response variables, and range (core or edge), collection site, and eclosion date were fixed effects. No random effects were included in the MANOVA.

Mass of female beetles at adult emergence was analyzed individually with a linear mixed model, with range as a fixed effect and collection site as a random effect.

Since some females (17%) did not lay eggs within 10 days, the data on the numbers of eggs were split into two datasets, one including the number of eggs for laying individuals, and the other including the binary response (laying or nonlaying) for all individuals, to assess whether probability of laying and fecundity differed between core and edge sites. To account for overdispersion, a negative binomial mixed model was fit to the count data (excluding nonlaying individuals) using the glmmTMB package version 1.0.2.1 (Brooks et al., 2017) with range, mass of beetle at emergence, and age at first reproduction as fixed effects and collection site as a random effect. A logistic mixed model was fit with the glmmTMB package to the binary dataset with range and mass as fixed effects and collection site as a random effect. Age at first reproduction could not be included as a covariate due to convergence issues.

Age at first reproduction was analyzed for individuals that reproduced during the experiment using a Conway‐Maxwell Poisson mixed model which accounts for under‐dispersion in this dataset (Brooks et al., 2019), with range and mass as fixed effects and collection site as a random effect. We complemented this analysis with a Kaplan–Meier survival analysis, which accounts for censoring of individuals that either did not reproduce during the experiment or died before reproducing. Since covariates and random effects cannot be added in Kaplan–Meier survival analyses, the analysis was run twice, once with collection site as the predictor to visualize the spread among sites and again with range as the predictor to estimate the total effect of range.

Mass of male beetles at the time of the dispersal trial was analyzed with a linear mixed model, with range, rearing density (low or high), mating status (unmated or mated), and interactions between those factors as fixed effects, age at time of weighing as a fixed covariate, and collection site as a random effect.

Each of the four dispersal elements (occurrence of flight, number of flights, total distance, and average speed) was analyzed separately. We chose not to do multivariate analyses on the dispersal traits since all but one variable was highly zero‐inflated and skewed, which violates assumptions of multivariate tests such as MANOVA, and univariate tests could better incorporate sampling design and environmental covariates during the trials, which greatly improve model fit. For each dispersal element, the same factors were included. Range, density, mating status, and all interactions were fixed effects. Mass at the time of dispersal trial, age, mill friction (Appendix S1), and air temperature were fixed covariates. Collection site and trial date were random effects.

The occurrence of at least one flight was analyzed with a binomial model with the packages lme4 version 1.1–26 (Bates et al., 2015) and lmerTest version 3.1–3 (Kuznetsova et al., 2017). The number of flights during the 1‐h trail was analyzed using both a negative binomial and a Poisson mixed model with the glmmTMB package. QQ plots of model residuals and residual vs. fitted plots from the DHARMa package version 0.3.3.0 were used to assess model fit (Hartig, 2020). The negative binomial mixed model best met assumptions of normality of residuals and was chosen as the final model. Total distance was a count of revolutions of the flight mill and thus a discrete variable. Six models were fit for total distance: a linear mixed model on log‐transformed distance, a negative binomial mixed model, a generalized Poisson model (commonly used for highly right‐skewed data with a high frequency of low counts (Brooks et al., 2019; Joe & Zhu, 2005)), and a zero‐inflated version of each of those, using the glmmTMB package. Based on the same residual diagnostics as above, the zero‐inflated generalized Poisson model best met assumptions of normality of residuals and was chosen as the final model. Average speed during the 1‐h trial included only trials in which the beetle made at least one flight and was analyzed with a linear mixed model using lme4 and lmerTest.

In all dispersal models, the three‐way interaction was dropped from the model if it was not statistically significant. Post hoc comparison of means was done with the emmeans package version 1.5.4 (Lenth, 2020). All analyses were done in R version 3.6.2 (R Core Team, 2018).

## 3 RESULTS

### 3.1 Life‐history

The MANOVA showed statistically significant differences in reproductive life‐history traits between core and edge, collection sites, and eclosion dates (range: Pillai–Bartlett statistic_1_ = 0.093, *p* < 0.001; collection site: Pillai–Bartlett statistic_6_ = 0.133, *p* = 0.046; eclosion date: Pillai–Bartlett statistic_3_ = 0.359, *p* < 0.001). Across all three traits, reproduction increased about 7% at the edge compared to core.

**FIGURE 3:**
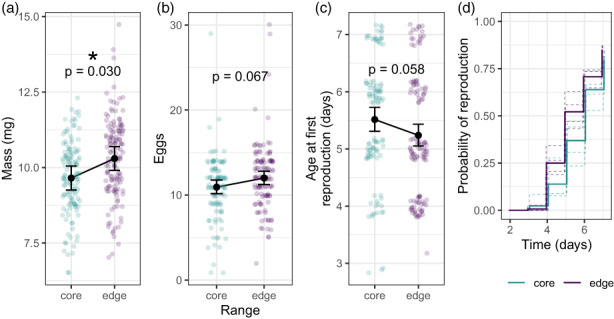
Means and 95% confidence intervals of the life‐history traits of female body mass (a), 24‐h fecundity (b), and age at first reproduction, analyzed with regression (c) and Kaplan–Meier survival analysis (d). Observations from laying individuals only are shown in b–c and are jittered to show individual points. In d, solid lines represent means of core and edge only and dashed lines are each population.

In the univariate analysis, mass of females at adult emergence ranged from 6.5 to 14.7 mg. Females from the edge of the range were larger than females from the core on average (core mass = 9.65 mg SE = 0.163; edge mass = 10.30 mg SE = 0.160; *F*_1_ = 8.04, *p* = 0.030; Figure 3a).

The number of eggs from the first day of reproduction ranged from 0 to 30. The proportion of beetles laying eggs during the study did not differ between the core and edge (core proportion = 0.813, edge proportion = 0.845, $\chi_{1}^{2}$ = 0.46, *p* = 0.499). Of those that oviposited during the study, edge beetles tended to be more fecund (core eggs = 10.9, SE = 0.40; edge eggs = 12.0, SE = 0.40; $\chi_{1}^{2}$ = 3.39, *p* = 0.067; Figure 3b). Age at first reproduction was not a predictor of the number of eggs laid ($\chi_{1}^{2}$= 0.01, *p* = 0.916), but larger females laid more eggs ($\chi_{1}^{2}$ = 5.79, *p* = 0.016).

Age at first reproduction ranged from 3 to 7 days after adult emergence. From the regression analysis, edge beetles tended to reproduce earlier than core beetles (core age = 5.51 days, SE = 0.11; edge age = 5.24 days, SE = 0.10; $\chi_{1}^{2}$ = 3.64, *p* = 0.058; Figure 3c). Mass at emergence was not a predictor of age at first reproduction ($\chi_{1}^{2}$= 1.92, *p* = 0.166). Results were similar in the Kaplan–Meier survival analysis. Median age at first reproduction was 5 days (95% CI: 5, 6) for edge and 6 days (95% CI: 6, 6) for core (Figure 3d). The log‐rank test for differences in survival curves indicated marginal differences between core and edge ($\chi_{1}^{2}$ = 3.8, *p* = 0.051).

The mass of males at the time of the dispersal trial (thus, after feeding ad libitum) ranged from 6.9 to 19.3 mg. There was no difference in mass of males between core and edge sites (*F*_1_ = 0.67, *p* = 0.445). Older beetles weighed more than younger beetles (*F*_1_ = 46.55, *p* < 0.001) and males reared at high density weighed more than those reared at low density (high mass = 12.2 mg, SE = 0.134; low mass = 11.8 mg, SE = 0.135; *F*_1_ = 15.09, *p* < 0.001).

### 3.2 Dispersal

All four dispersal elements were positively correlated with each other (Spearman rank‐order correlation, all pairwise comparisons *p* < 0.05). In the statistical models, the three‐way interaction between range, density, and mate status was not statistically significant in any model, so it was removed from all models. Weight was significantly positively associated with all four dispersal elements (Table S2). Results for covariates and random effects are in Table S2.

**FIGURE 4:**
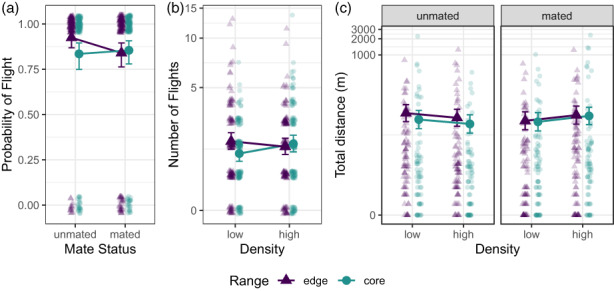
Means and 95% confidence intervals of dispersal elements. Mating status‐dependent dispersal evolved from core to edge for probability of flight (a), and density‐dependent dispersal evolved from core to edge for number of flights (b). Flight distance is informed by mating status and density (c). Observations have been jittered to show individual points. Note log‐scale *y*‐axis in b and c.

During the dispersal trials, 84.2% of all beetles took at least one flight. For occurrence of flight, the interaction between mating status and range was statistically significant ($\chi_{1}^{2}$ = 4.14, *p*‐value = 0.042), indicating unmated edge beetles were more likely to fly than unmated core beetles (core probability = 0.84, edge probability = 0.93; *z* = −2.309, *p* = 0.021), but there was no difference for mated beetles (core probability = 0.86, edge probability = 0.84; *z* = 0.356, *p* = 0.722; Figure 4a). The interaction between density and mate status was close to statistical significance ($\chi_{1}^{2}$ = 3.01, *p* = 0.083), such that unmated beetles tended to fly more than mated beetles at low density, but there was no difference between mated and unmated at high density (Figure S2A).

The number of flights during the 1‐h trial ranged from 0 to 13 (median = 2). The interaction between density and range was statistically significant ($\chi_{1}^{2}$ = 4.93, *p* = 0.026), indicating at low density, edge beetles took more flights than core beetles (core flights = 1.68, SE = 0.15; edge flights = 2.13, SE = 0.17; *t*_576_ = −2.064, *p* = 0.040), but there was no difference at high density (core flights = 2.03, SE = 0.16; edge flights = 1.93, SE = 0.15; *t*_576_ = 0.490, *p* = 0.625; Figure 4b). The interaction between density and mate status was close to statistical significance, demonstrating that unmated beetles tended to take more flights than mated beetles at low density and the opposite at high density ($\chi_{1}^{2}$ = 3.48, *p* = 0.062) (Figure S2B).

Individuals flew up to 2.3 km (median = 8 m), during the 1‐h trial. The interaction between density and mate status was statistically significant in the final model, such that unmated beetles flew further than mated beetles at low density, but the opposite at high density ($\chi_{1}^{2}$ = 6.13, *p* = 0.013; Figure 4c). The range main effect was also close to statistical significance, indicating edge beetles tended to fly further than core beetles (core distance = 59.6 m, SE = 10.01; edge distance = 69.5, SE = 11.45; $\chi_{1}^{2}$= 2.998, *p* = 0.083), and this difference was most pronounced for unmated beetles at low density.

Average flight speed ranged from 0.138 to 0.914 m/s and did not differ by range, density, mate status or their interactions (Figure S2C).

## 4 DISCUSSION

Theory predicts that both life‐history and dispersal traits will evolve during range expansion (e.g. Peischl et al., 2015; Peischl & Excoffier, 2015; Phillips et al., 2010b; Shine et al., 2011), with different evolutionary processes driving distinct patterns of evolution. It is unclear how well theoretical predictions hold up in complex natural systems including biocontrol agents, and which evolutionary processes appear to drive trait changes across range expansions (Szűcs et al., 2019). We studied the recent range expansion of the biocontrol agent *D. carinulata* in the western US and infer the dominant evolutionary processes acting during the range expansion by comparing life‐history and dispersal traits from individuals at the core and edge of the range.

A trend towards higher fecundity and earlier age at first reproduction in individuals from the edge suggests that selection at low densities dominates over expansion load in driving evolution of reproductive life‐history traits at the edge of the range. Females from the edge laid on average only one more egg than those from the core on the first day of reproduction. While this pattern was only weakly supported statistically, the biological effects could be large. If this difference persists throughout the multi‐week lifespan of adults, it could sum to a substantial difference in fitness between core and edge individuals. Additionally, early fecundity in *D. carinulata* is a good predictor of lifetime fecundity (Bitume et al., 2017). Edge females also reproduced earlier than core beetles. Earlier age at first reproduction is a trait classically associated with “r” selection of low‐density environments (Stearns, 1992). Age at first reproduction is an often‐overlooked trait that can be just as important for fitness as fecundity itself, since reproducing earlier can increase the total time available for an individual to reproduce and early offspring often have an advantage over later offspring because there are fewer other offspring to compete with (Stearns, 1976). Adults reaching reproductive maturity faster could allow more generations per year, which might allow acceleration of the range expansion from edge populations of *D. carinulata*, which are also less constrained by cold temperatures in the winter (Jamison et al., 2018). We used mass as an additional gauge of fecundity, since insect body size is often related to egg production and how many eggs females can carry (Berger et al., 2012). We found this association between mass and fecundity to hold in *D. carinulata* and that individuals from the edge were larger than those from the core. Higher fecundity, earlier age at first reproduction, and larger mass of females all suggest that selection has increased reproductive capacity at the edge of the range expansion. While the effect sizes are small for each individual trait, when viewed together, this provides strong evidence for a selection‐driven shift in reproductive life‐history traits across the range about 15 years after initial biocontrol releases.

These same phenotypic patterns provide evidence against the evolution of increased genetic load (or reduced fitness) in edge sites relative to core sites. Recent evidence of high genetic diversity along one *D. carinulata* expansion front (Stahlke et al., 2022) is consistent with no measurable genetic load, as the deleterious alleles responsible for genetic load are likely at low frequency in diverse populations. Low genetic load and high genetic variation among *D. carinulata* may also be explained by the prerelease history of this biocontrol agent. Specifically, they were collected from multiple source populations and population sizes were deliberately large to avoid reducing variation that could increase establishment in the field (Stahlke et al., 2022; Szűcs et al., 2017).

Dispersal is an inherently and notoriously variable behavioral trait (Bowler & Benton, 2005), and we found this to be true for *D. carinulata*, even when measuring dispersal in a controlled lab environment. Accounting for the density and mating context of dispersal decisions in our experiments allowed us to observe context‐dependent evolution between core and edge more clearly and test hypotheses about the mechanisms behind the patterns we see. The occurrence of flight was affected by mating status along the range expansion such that unmated beetles from the edge flew more than those from the core. This implies that the response to being unmated has evolved between core and edge. Males on the edge may have evolved this behavior if mate availability is often low at the edge. For the number of flights, dispersal became negatively density‐dependent at the edge, such that the number of flights increased in low‐density environments compared to core, while staying about the same in high‐density environments. This implies that the response to density has evolved during range expansion. Evolution of the response to density could be due to selection for increased dispersal at range edges at low density, as predicted by theory (De Bona et al., 2019; Fronhofer, Nitsche, & Altermatt, 2017; Travis et al., 2009). Estimates of beetle density in the field would be valuable for further exploring this mechanism. However, accurately measuring beetle density in the field is not without challenges, as density can vary within a collection site due to many factors, including beetle life stage, ephemeral weather patterns, and plant health (Henry et al., 2018), as well as range expansion.

Edge beetles flew further than core beetles across all density and mating treatments, though this pattern was most pronounced for unmated beetles at low density, as expected by condition‐dependent dispersal theory. Unlike with occurrence of flight and number of flights, the relationship of distance flown with mating status and density did not change over the range, but we do find a weak signature of spatial sorting of dispersal ability. In this species, spatial sorting might primarily act on occurrence or frequency of flights rather than flight distance or speed if most dispersal flights driving the range expansion are comprised of multiple frequent flights to catch air currents, instead of long‐distance flights. Future studies will be needed to explore how *D. carinulata* disperse in nature and how spatial sorting acts on different dispersal elements in natural systems.

The effect of spatial sorting in the range expansion of *D. carinulata* could be small because of maladaptation to novel environments on the edge of the range expansion that slow down range expansion and reduces assortative mating between dispersive individuals at the edge (Andrade‐Restrepo et al., 2019; Hillaert et al., 2015). Early in its range expansion, *D. carinulata* was maladapted to photoperiod cues (Bean et al., 2007a, 2012) and possibly higher summer temperatures in southern latitudes (Herrera et al., 2005). Adaptation to photoperiod has limited the rate of southern range expansion in this beetle and thus may reduce the effect of spatial sorting of dispersal. Despite this, our results suggest that spatial processes during range expansion may be important to natural range expansions even over heterogenous environments.

In many species, dispersal evolves along with suites of traits, called dispersal syndromes (Ronce & Clobert, 2012), and in some cases, many life‐history traits may correlate well with dispersal (Stevens et al., 2013). Trade‐offs between dispersal and reproductive ability are widely hypothesized to be present due to allocation of finite resources (Bonte & Dahirel, 2017; Stearns, 1989) though support for such trade‐offs during range expansion is mixed (e.g. Hughes et al., 2003; Jan et al., 2019; Kelehear & Shine, 2020; Tabassum & Leishman, 2018; Therry et al., 2015). In the *D. carinulata* range expansions, we do not see evidence of a trade‐off between dispersal and life‐history traits, though we were unable to measure all traits within the same individuals. One hypothesis for how this species has apparently increased dispersal and avoided genetic load is due to their ability to aggregate using a male‐produced pheromone (Bean, Wang, et al., 2007; Cossé et al., 2005). While the expanding edge is led by individuals that are superior dispersers (thus allowing for spatial selection on dispersal), they can use pheromones to attract others to the area and population sizes can quickly increase, thus decreasing the negative effects of small population sizes. We measured dispersal for only male *D. carinulata*, but there are many reasons for dispersal to differ between the sexes (reviewed in Li & Kokko, 2019). There may also be trade‐offs between dispersal and other traits, such as lifespan or immune system development or function (reviewed in Chuang & Peterson, 2016).

Long‐term success of the *Tamarix‐Diorhabda* biocontrol program requires *D. carinulata* to continue its spread to cover the range of the target weed and to adapt to new environments. We show an increase in both reproductive output and dispersal ability in some contexts at the edge and low genetic load, which may enable an accelerating expansion front and will likely contribute to the establishment and persistence of *D. carinulata* populations at the edge (Phillips et al., 2010a). Evolution of these traits and others previously studied (Bean et al., 2012; Stahlke et al., 2022) suggests that there is sufficient genetic variation for populations to continue to adapt to novel environments during the expansion. As the first test of evolutionary theory of range expansions in a modern biocontrol agent, we show that these theoretical predictions can be applied to range expansions across heterogeneous environments, especially when the ecological context of individuals is included. We may expect to find selection at low densities to be the dominant evolutionary process over expansion load and for spatial sorting to act on other biocontrol agents that share many characteristics with *D. carinulata* (e.g. Bartelt et al., 2008; Muller‐Scharer et al., 2014). Our results suggest that evolutionary processes impacting range expansions of natural populations can act simultaneously with adaptation to environmental gradients.

## CONFLICT OF INTEREST

The authors declare no conflict of interests.

## Supporting information


Appendix S1

Appendix S2

## Data Availability

Data from this study are available at Ag Data Commons: https://doi.org/10.15482/USDA.ADC/1522916.
