# Supplementary material for: Evolution of reproductive life‐history and dispersal traits during the range expansion of a biological control agent
Source: Evol Appl. 2022 Nov 1;15(12):2089–99. doi: 10.1111/eva.13502 (PMC9753830; doi:10.1111/eva.13502)
Supplement: Supplementary file 1 — Appendix S1 [file EVA-15-2089-s002.docx]

**Supplementary Material**

**Table S1: Collection sites**

**Table S1.** Collection sites for the eight populations used in the life history and dispersal studies.

|  | Population | Range | Latitude | Longitude | Collection Year |
| --- | --- | --- | --- | --- | --- |
| A | Lovell, Wyoming | Core | 44.856 | -108.207 | 2017 |
| B | Humboldt, Nevada | Core | 40.063 | -118.590 | 2018 |
| C | Delta, Utah | Core | 39.144 | -112.958 | 2018 |
| D | Pueblo, Colorado | Core | 38.268 | -104.721 | 2018 |
| E | Blythe, California | Edge | 33.912 | -114.533 | 2018 |
| F | Wickenburg, Arizona | Edge | 34.422 | -112.701 | 2018 |
| G | Little Colorado River, Arizona | Edge | 34.593 | -109.611 | 2018 |
| H | La Joya, New Mexico | Edge | 34.342 | -106.864 | 2017 |

**Appendix S1: Dispersal Trials**

**Figure S1.** Diagram of flight mills used in dispersal trials. **A.** Low friction plastic base with tiny hole for wire to freely rotate. **B.** Teflon-coated wire. **C.** Paper flag to block sensor each rotation. **D.** Infra-red light emitter. **E.** Infra-red light sensor. **F.** Poster putty beetle attachment. Each flight mill was also equipped with a light source and a heat source.

**
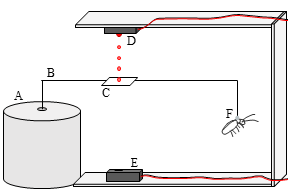
**

Flight activity in *D. carinulata* has been observed in the field to occur primarily in the afternoon and at high temperatures, so trials were conducted between 7 and 11 hours after lights on and heating cables were used to increase the air temperature during flight trials. Actual air temperature during each trial was recorded using HOBO sensors.

For the dispersal trials, a beetle was attached by the pronotum to the wire of the flight mill with a small piece of poster putty. Beetles started the trial holding on to a small strip of paper to act as a support. The strip of paper was returned to the beetle between flights during the trial. The paper was dropped when the beetle initiated a flight, so it did not impact the weight of the beetle during flight.

Differences in friction between the 24 flight mill set-ups were evaluated by applying a standardized force to the wire of each flight mill and calculating the ratio of the duration of the first rotation to the duration of the second rotation. A higher ratio indicates less friction in the flight mill.

**Appendix S3: Dispersal Statistical Results**

**Figure S2.** Results of all two-way interactions between range, density, and mating status for occurrence of flight (**A**), number of flights (**B**), and average flight speed (**C**).


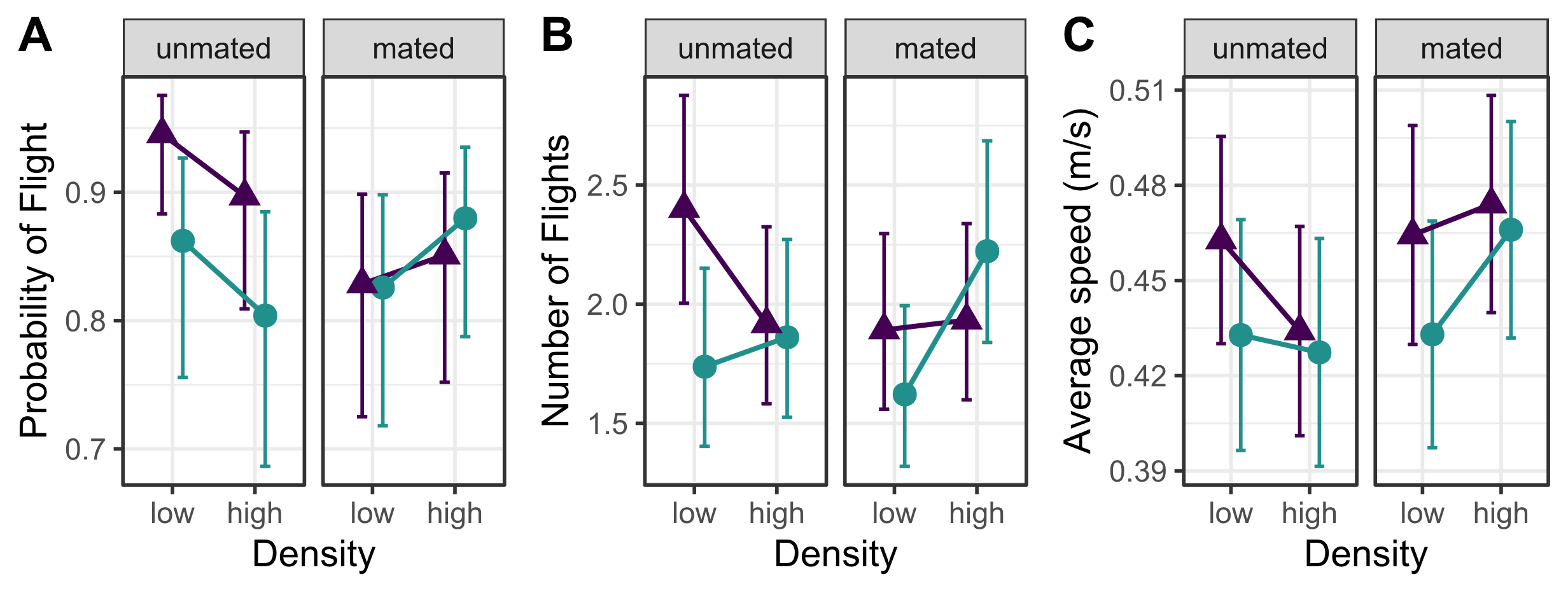


**Table S2.** Results from the final model for each of the four dispersal parameters. Shown are the variance of random effects, and Χ^2^ or F-value and p-value for all main effects, interactions, and covariates.

|  | **Probability of flight** | | | **Number of flights** | | |
| --- | --- | --- | --- | --- | --- | --- |
| Type of model: | Binomial | | | Negative binomial | | |
|  | Var | Χ^2^ | p | Var | Χ^2^ | p |
| Density |  | 1.01 | 0.315 |  | 0.33 | 0.567 |
| Mate status |  | 0.44 | 0.507 |  | 0.32 | 0.574 |
| Range |  | 4.61 | **0.032** |  | 5.94 | **0.015** |
| Density*Mate |  | 3.01 | 0.083 |  | 3.48 | 0.062 |
| Density*Range |  | 0.30 | 0.582 |  | 4.93 | **0.026** |
| Mate*Range |  | 4.14 | **0.042** |  | 1.63 | 0.202 |
| Weight |  | 13.55 | **0.000** |  | 11.64 | **0.001** |
| Age |  | 1.31 | 0.253 |  | 1.11 | 0.293 |
| Mill friction |  | 1.77 | 0.184 |  | 1.16 | 0.281 |
| Temperature |  | 9.70 | **0.002** |  | 5.11 | **0.024** |
| Population (R) | 0.00 | 0.00 | 1.000 | 0.01 | 0.00 | 1.000 |
| Trial date (R) | 0.10 | 1.72 | 0.189 | 0.00 | 0.05 | 0.829 |
|  | **Flight distance** | | | **Flight speed** | | |
| Type of model: | Generalized Poisson | | | Linear | | |
|  | Var | Χ^2^ | p | Var | F | p |
| Density |  | 1.38 | 0.240 |  | 0.03 | 0.867 |
| Mate status |  | 0.36 | 0.550 |  | 2.55 | 0.111 |
| Range |  | 3.00 | 0.083 |  | 2.14 | 0.196 |
| Density*Mate |  | 6.13 | **0.013** |  | 2.39 | 0.123 |
| Density*Range |  | 0.00 | 0.956 |  | 0.88 | 0.348 |
| Mate*Range |  | 1.69 | 0.193 |  | 0.00 | 0.956 |
| Weight |  | 21.73 | **0.000** |  | 12.26 | **0.001** |
| Age |  | 0.11 | 0.736 |  | 10.10 | **0.005** |
| Mill friction |  | 3.12 | 0.077 |  | 0.84 | 0.359 |
| Temperature |  | 0.48 | 0.489 |  | 0.57 | 0.449 |
| Population (R) | 0.00 | 0.16 | 0.687 | 0.00 | 0.00 | 1.000 |
| Trial date (R) | 0.04 | 8.73 | 0.003 | 0.00 | 0.38 | 0.536 |
