# Supplementary material for: Evolution of reproductive life‐history and dispersal traits during the range expansion of a biological control agent
Source: Evol Appl. 2022 Nov 1;15(12):2089–99. doi: 10.1111/eva.13502 (PMC9753830; doi:10.1111/eva.13502)
Supplement: Supplementary file 2 — Appendix S2 [file EVA-15-2089-s001.docx]

**Appendix S2:** **Data Analysis Scripts**

Python script for flight mill sensor analysis. This program was used to analyze the raw sensor output to generate summary statistics for each trial, including number of flights, total distance, and average flight speed.

#!/usr/bin/env python3

# Tested with Python 3.5

from datetime import datetime

import itertools

from bisect import bisect

from collections import (

deque,

namedtuple,

)

import csv

from pathlib import Path

"""

Each flight the longest-possible contiguous subsequence sequence of laps in

which the beetle spent between MIN_SENSOR_TIME and MAX_SENSOR_TIME seconds

under the sensor followed by MIN_INTERMEDIATE_TIME seconds away from the

sensor, with the total lap time under MAX_LAP_TIME seconds.

"""

MIN_SENSOR_TIME = 0.045

MAX_SENSOR_TIME = 0.65

MIN_INTERMEDIATE_TIME = 0.8

MAX_LAP_TIME = 7.8

# LAP_METERS is the circumference of the flight mill in meters.

LAP_METERS = 1

# Metadata contains trial metadata parsed from a filename.

Metadata = namedtuple('Metadata', [

'trial_date',

'labjack',

'mill',

'run',

])

# SensorCrossing contains the start and end times of triggering the sensor.

SensorCrossing = namedtuple('SensorCrossing', [

'start',

'end',

])

# Trial contains trial summary statistics.

Trial = namedtuple('Trial', [

'metadata',

'start_time',

'end_time',

'num_flights',

'distance',

'duration',

'average_speed',

'flights',

'flew',

])

# Flight contains flight statistics.

Flight = namedtuple('Flight', [

'start_time',

'distance',

'average_speed',

'duration',

'max_speed',

'min_speed',

'endurance',

])

def parse_filename(filename):

"""

Parses Metadata from a CSV filename.

"""

date_raw, labjack, mill_run_str = Path(filename).stem.split(sep = '_', maxsplit = 2)

date_pretty = '20{}-{}-{}'.format(date_raw[4:], date_raw[:2], date_raw[2:4])

mill_run = mill_run_str.replace('-','_').split('_')

mill = mill_run[0]

run = 1 if len(mill_run) < 2 else int(mill_run[1])

return Metadata(date_pretty, labjack, mill, run)

def sensor_crossings(rows):

"""

Returns an iterable of the SensorCrossing objects showing when the sensor was triggered.

"""

COL_TIMESTAMP = 0

COL_SENSOR = 1

start_time = None

for key, group in itertools.groupby(rows, lambda row: row[COL_SENSOR]):

# Take the first timestamp from each span of zeroes.

if key == 0:

start_time = next(group)[COL_TIMESTAMP]

elif key == 1 and start_time != None:

yield SensorCrossing(start=start_time,

end=next(group)[COL_TIMESTAMP])

def _min_min_max_valid_lap(min_sensor, max_sensor, min_inter, max_lap, crossing1, crossing2):

sensor_time = crossing1.end - crossing1.start

lap_time = crossing2.start - crossing1.start

inter_time = lap_time - sensor_time

return sensor_time >= min_sensor and sensor_time <= max_sensor and inter_time >= min_inter and lap_time <= max_lap

def min_min_max_starter(min_sensor, max_sensor, min_inter, max_total):

def min_min_max_starter_1(crossings):

crossings_iter = iter(crossings)

last_crossing = None

for crossing in crossings_iter:

if last_crossing is not None and _min_min_max_valid_lap(min_sensor, max_sensor, min_inter, max_total, last_crossing, crossing):

yield last_crossing

yield crossing

break

last_crossing = crossing

yield from crossings_iter

return min_min_max_starter_1

def min_min_max_splitter(min_sensor, max_sensor, min_inter, max_total):

def min_min_max_splitter_1(crossings):

crossings_iter = iter(crossings)

last_crossing = None

flight = deque()

for crossing in crossings_iter:

if last_crossing is None or _min_min_max_valid_lap(min_sensor, max_sensor, min_inter, max_total, last_crossing, crossing):

flight.append(crossing)

last_crossing = crossing

else:

return (list(flight), itertools.chain([crossing], crossings_iter))

return (list(flight) if len(flight) > 0 else None, None)

return min_min_max_splitter_1

def extract_flights(starter, splitter, timestamps):

"""

Returns an iterator of iterators of timestamps for each long flight.

`starter` is a function, such as the result of `min_min_max_starter`, to

consume timestamps until the beginning of a flight. `splitter` is a function

such as `min_min_max_splitter` to split the end of a flight from the rest of

timestamps.

"""

while timestamps != None:

timestamps = starter(timestamps)

flight, timestamps = splitter(timestamps)

if flight != None:

yield flight

def flight_endurance(crossings):

"""

Returns the distance flown in the first half of a flight

divided by the distance flown in the second half.

The flight is split at the lap boundary closest to half of

the total flight duration.

"""

timestamps = list(map(lambda cr: cr.start, crossings))

if len(timestamps) < 3:

return None

halftime = (timestamps[0] + timestamps[-1]) / 2

index_after = bisect(timestamps, halftime)

index_before = index_after - 1

lap_elapsed_before = halftime - timestamps[index_before]

lap_elapsed_after = timestamps[index_after] - halftime

laps_by_halftime = index_after if lap_elapsed_after < lap_elapsed_before else index_before

total_laps = len(timestamps) - 1

endurance = laps_by_halftime / (total_laps - laps_by_halftime)

return endurance

def analyze_flight(crossings):

"""

Returns a dict of statistics on the flight described by the given iterable

of sensor crossings.

"""

start_time = datetime.utcfromtimestamp(crossings[0].start)

lap_count = len(crossings) - 1

distance = lap_count * LAP_METERS

duration = crossings[-1].start - crossings[0].start

average_speed = distance / duration if duration != 0 else ''

endurance = flight_endurance(crossings)

min_speed = average_speed

max_speed = average_speed

for i in range(lap_count):

lap_speed = LAP_METERS / (crossings[i+1].start - crossings[i].start)

min_speed = min(lap_speed, min_speed)

max_speed = max(lap_speed, max_speed)

return Flight(

start_time = start_time,

distance = distance,

average_speed = average_speed,

duration = duration,

max_speed = max_speed,

min_speed = min_speed,

endurance = endurance

)

def analyze_file(filename):

"""

Returns a dict of trial statistics computed from the given file.

"""

metadata = parse_filename(filename)

# Read sensor timestamps from CSV file

row_to_floats = lambda row: list(map(float, row))

with open(filename, newline = '') as file:

reader = csv.reader(file)

# Skip the header row

next(reader)

rows = list(map(row_to_floats, reader))

crossings = list(sensor_crossings(rows))

# Do the actual work

all_flight_stats = []

distance = 0

duration = 0

starter = min_min_max_starter(MIN_SENSOR_TIME, MAX_SENSOR_TIME, MIN_INTERMEDIATE_TIME, MAX_LAP_TIME)

splitter = min_min_max_splitter(MIN_SENSOR_TIME, MAX_SENSOR_TIME, MIN_INTERMEDIATE_TIME, MAX_LAP_TIME)

for flight in extract_flights(starter, splitter, crossings):

flight_stats = analyze_flight(flight)

distance += flight_stats.distance

duration += flight_stats.duration

all_flight_stats.append(flight_stats)

average_speed = distance / duration if duration != 0 else ''

start_time = datetime.utcfromtimestamp(rows[0][0]) if len(rows) > 0 else ''

end_time = datetime.utcfromtimestamp(rows[-1][0]) if len(rows) > 0 else ''

return Trial(

start_time = start_time,

end_time = end_time,

num_flights = len(all_flight_stats),

distance = distance,

duration = duration,

average_speed = average_speed,

flights = all_flight_stats,

flew = 'yes' if len(all_flight_stats) > 0 else 'no',

metadata = metadata

)

def write_data(trials_stats, trials_filename, flights_filename):

"""

Writes data from a dict of filename and trials statistics to two CSV files

with the given names.

"""

def openfile(name):

return open(name, 'w', newline='')

#meta_fields = ['trial_date', 'labjack', 'mill', 'run']

meta_fields = list(Metadata._fields)

with openfile(trials_filename) as tfile, openfile(flights_filename) as ffile:

twriter = csv.DictWriter(tfile, meta_fields + ['start_time', 'end_time', 'num_flights', 'distance', 'duration', 'average_speed', 'flew'], extrasaction='ignore')

fwriter = csv.DictWriter(ffile, meta_fields + ['flight_num', 'start_time', 'distance', 'duration', 'average_speed', 'max_speed', 'min_speed', 'endurance'])

twriter.writeheader()

fwriter.writeheader()

for filename, trial in trials_stats.items():

twriter.writerow(dict(**trial._asdict(), **trial.metadata._asdict()))

flights = trial.flights

for i in range(len(flights)):

labeled_flight = dict(**trial.metadata._asdict(), flight_num = i+1, **flights[i]._asdict())

fwriter.writerow(labeled_flight)

def inputdefault(prompt, default):

"""

Equivalent to input(), but displays a default value and returns it

if the user provides an empty string.

"""

s = input('{} (default = "{}"): '.format(prompt, default))

if s == '':

return default

else:

return s

if __name__ == '__main__':

dirname = inputdefault('Directory with CSV files', '.')

trials_filename = inputdefault('Trials output file', 'trials.csv')

flights_filename = inputdefault('Flights output file', 'flights.csv')

trials = {}

p = Path(dirname)

for child in p.iterdir():

if Path(child).suffix.lower() == '.csv':

trials[str(child)] = analyze_file(str(child))

write_data(trials, flights_filename = flights_filename, trials_filename = trials_filename)
